# Suggestive associations between genetically predicted gut microbiota and endometriosis: a two-sample Mendelian randomization study

**DOI:** 10.1099/jmm.0.002174

**Published:** 2026-07-01

**Authors:** Liyun Liao, Zhixiang Zhou, Xiaoyang Ye, Yi Lin, Panpan Hu, Qiaoyun Zhang, Zhifen Wang, Dandan Yang, Huan Lu, Minna Wang, Jianshuo Lu

**Affiliations:** 1 Gynecology, Xiangshan First People’s Hospital Medical and Health Group, Ningbo City, Zhejiang Province, 315700, PR China; 2 Emergency Department, Xiangshan First People’s Hospital Medical and Health Group, Ningbo City, Zhejiang Province, 315700, PR China; 3 Vascular Surgery, Xiangshan First People’s Hospital Medical and Health Group, Ningbo City, Zhejiang Province, 315700, PR China

**Keywords:** endometriosis, gastrointestinal microbiome, genome-wide association study, Mendelian randomization analysis

## Abstract

**Introduction.** Endometriosis affects 10–20% of reproductive-age women. Emerging evidence links the gut microbiota to endometriosis pathogenesis, but observational studies are limited by confounding, reverse causation and uncertainty about whether reported microbial signals represent reproducible aetiological associations.

**Hypothesis/Gap Statement.** Whether genetically predicted gut microbial genera are associated with endometriosis risk remains unclear, and available observational evidence does not establish robust causal effects after accounting for multiple testing.

**Aim.** This study aimed to explore potential Mendelian randomization (MR)-based associations between genetically predicted gut microbiota composition and endometriosis using a two-sample MR framework.

**Methodology.** Genome-wide association study (GWAS) summary statistics for 119 bacterial genera (MiBioGen consortium: *n*=18,340) and endometriosis (FinnGen: 8,288 cases, 68,969 controls) were used. Single nucleotide polymorphisms (SNPs) associated with each genus (*P*<5×10⁻⁵) were selected as instrumental variables after linkage disequilibrium clumping and weak instrument exclusion. The primary method was inverse-variance weighting (IVW), supplemented by four complementary methods. Benjamini–Hochberg false discovery rate (FDR) correction was applied across all 119 genera.

**Results.** Seven genera showed nominally significant IVW associations with endometriosis (*P*<0.05): *Lactococcus*, *Olsenella*, *Senegalimassilia*, Ruminococcaceae UCG-002, *Holdemania*, *Eubacterium ruminantium* group and *Anaerotruncus*. *Olsenella*, Ruminococcaceae UCG-002 and *Anaerotruncus* were directionally associated with increased risk, whereas the remaining four were directionally associated with reduced risk. However, none survived FDR correction (all FDR-adjusted *P*=0.731). No significant heterogeneity or horizontal pleiotropy was detected.

**Conclusion.** These findings provide exploratory and suggestive MR evidence for potential associations between specific gut microbial genera and endometriosis, rather than definitive causal evidence. As no associations survived multiple testing correction, these results should be interpreted as hypothesis-generating and require replication in larger, ancestry-matched cohorts.

## Data Summary

The data that support the findings of this study are available from the corresponding author upon reasonable request.

## Introduction

Endometriosis is a common gynaecological disorder predominantly affecting women of reproductive age, clinically characterized by secondary dysmenorrhoea, infertility, chronic pelvic pain, dyspareunia and menstrual abnormalities. This condition involves the ectopic implantation of endometrial glands and stroma, most frequently occurring in the ovaries and pelvic cavity. Epidemiological studies indicate a prevalence of 10–20% in the general female population, rising to ~50% among individuals with infertility [1,2]. Although histologically benign, endometriosis exhibits malignant-like invasive behaviour and high recurrence rates, correlated with dysmenorrhoea severity, disease stage, concurrent infertility and postoperative medication regimens [3]. Currently, hormonal drugs remain the primary treatment for controlling endometriotic lesion growth; however, long-term use is associated with various side effects [4]. Multiple hypotheses have been proposed to explain the pathogenesis of endometriosis, yet no definitive aetiology comprehensively accounts for its development [5].

Recent advances in high-throughput sequencing have revealed distinct compositional and abundance profiles in the gut microbiota of endometriosis patients compared with unaffected individuals. Concurrent serum analyses demonstrate elevated oestrogen and pro-inflammatory cytokine levels in patients, with these perturbations correlating with specific microbial signatures [6]. Experimental studies demonstrated that oral administration of broad-spectrum antibiotics or metronidazole reduced ectopic lesion burden in murine endometriosis models, whereas faecal microbiota transplantation restored lesion growth and pelvic inflammatory responses [7,8]. Furthermore, gut microbiota-derived short-chain fatty acids, particularly butyrate, suppressed the growth of both mouse ectopic lesions and human endometriotic lesions, and gut microbiota-derived metabolites may promote endometriosis progression through immune cell adaptation [8,9]. These findings substantiate a meaningful association between the gut microbiota and endometriosis pathogenesis.

However, inherent limitations in conventional epidemiological approaches – including confounding factors and reverse causality – contribute to persistent controversies. Mendelian randomization (MR) analysis, an instrumental variable approach leveraging genetic variants, can strengthen aetiologic inference when its core assumptions are satisfied [10]. Genetic variants function as instrumental variables because their prenatal random allocation follows Mendelian inheritance laws, conferring relative stability against postnatal environmental confounders [11]. The proliferation of large-scale genome-wide association study (GWAS) summary statistics has accelerated MR applications, generating evidence for aetiological investigations of common diseases [12]. Therefore, this study employed two-sample MR analysis as an exploratory, hypothesis-generating framework to evaluate potential genetic associations between the gut microbiota and endometriosis.

## Methods

### Data sources

The gut microbiota GWAS summary statistics were derived from a large-scale, multi-ethnic meta-analysis conducted by the MiBioGen consortium, encompassing 24 cohorts and 18,340 participants [13]. This dataset characterized gut microbial composition through targeted sequencing of hypervariable regions (V4, V3–V4 and V1–V2) of the 16S rRNA gene. All datasets were rarefied to 10,000 reads per sample to account for sequencing depth heterogeneity. Bacterial taxa were classified using direct taxonomic profiling, followed by quality control filters retaining taxa present in more than 10% of samples within each cohort, requiring an effective sample size exceeding 3,000, and restricting analysis to features detectable in at least three independent cohorts. Ultimately, 211 bacterial taxa were retained for downstream analyses. After adjusting for age, sex, technical covariates and genetic principal components, GWAS were performed on these taxa [13]. The GWAS summary statistics are publicly available at http://www.mibiogen.org.

The endometriosis GWAS dataset (finn-b-N14_ENDOMETRIOSIS) was sourced from the FinnGen biobank (https://www.finngen.fi/en), comprising summary statistics for 8,288 cases and 68,969 controls across 16,377,306 single nucleotide polymorphisms (SNPs). This dataset exclusively utilizes aggregated statistics and predominantly originates from European-ancestry populations. Because the MiBioGen consortium is a multi-ethnic dataset and the FinnGen endometriosis dataset comprises exclusively European-ancestry individuals, ancestry mismatch was considered a major interpretive limitation throughout the analysis and discussion.

### Instrumental variable selection

This MR analysis focused on bacterial taxa at the genus level. Instrumental variables were selected according to the following sequential criteria. First, SNPs were filtered at a significance threshold of *P*<5×10⁻⁵. This relaxed threshold, rather than the conventional genome-wide significance level of *P*<5×10⁻⁸, was adopted because gut microbiota GWAS currently yield very few genome-wide significant loci due to the polygenic nature and modest heritability of microbial traits, consistent with previous MR studies of gut microbiota [14]. Second, SNPs exhibiting allele mismatches (e.g. A/G vs. A/C) were excluded. Third, palindromic SNPs (A/T or G/C variants) were removed due to strand ambiguity. Fourth, SNPs in linkage disequilibrium were excluded using an r² < 0.01 and a genetic distance >10,000 kb, estimated based on the European population reference panel from the 1,000 Genomes Project. Fifth, F-statistics were calculated for each IV; instruments with F<10 were excluded to minimize weak instrument bias [15]. After these filtering steps, 119 bacterial genera were retained for analysis.

To assess the sensitivity of results to the SNP selection threshold, supplementary analyses were conducted using a more stringent threshold of *P*<1×10⁻⁵ for genera that retained a sufficient number of independent instruments (≥3 SNPs with F>10) at this level.

### MR analysis workflow

MR analyses were conducted using R version 4.4.0 with the ‘TwoSampleMR’ package, supplemented by Mendelian Randomization Pleiotropy RESidual Sum and Outlier (MR-PRESSO) analyses via the ‘MRPRESSO’ package. The analytical workflow comprised four components.

MR effect estimation: Primary MR-based association estimates employed inverse-variance weighting (IVW) [16], supplemented by MR-Egger regression [17], weighted median [18], simple mode and weighted mode approaches. IVW estimates were prioritized when no evidence of horizontal pleiotropy was detected [19].

Multiple testing correction: To account for multiple comparisons across 119 bacterial genera, Benjamini–Hochberg false discovery rate (FDR) correction was applied to the IVW *P*-values [20]. Associations with nominal *P*<0.05 but FDR-adjusted *P*≥0.05 were considered suggestive rather than statistically significant.

Statistical heterogeneity: Cochran’s Q test assessed heterogeneity across SNPs, with *P*≤0.05 indicating significant heterogeneity [21].

Horizontal pleiotropy and sensitivity analysis: Horizontal pleiotropy was evaluated through MR-Egger intercept analysis and the MR-PRESSO global test, with *P*>0.05 indicating no statistical evidence of directional pleiotropy. Funnel plots were examined for evidence of asymmetry. Leave-one-out analysis determined the influence of individual SNPs on IVW estimates, where stable effect sizes upon sequential SNP exclusion indicated limited dependence on any single variant [22].

## Results

### Determination of instrumental variables

Following exclusion of weak instruments, 119 bacterial genera were retained as exposure factors for MR analysis. All instrumental variables exhibited F-statistics>10 across these genera, with detailed characteristics provided in Table S1, available in the online Supplementary Material.

### MR results for endometriosis-associated gut microbiota

**Fig. 1.**
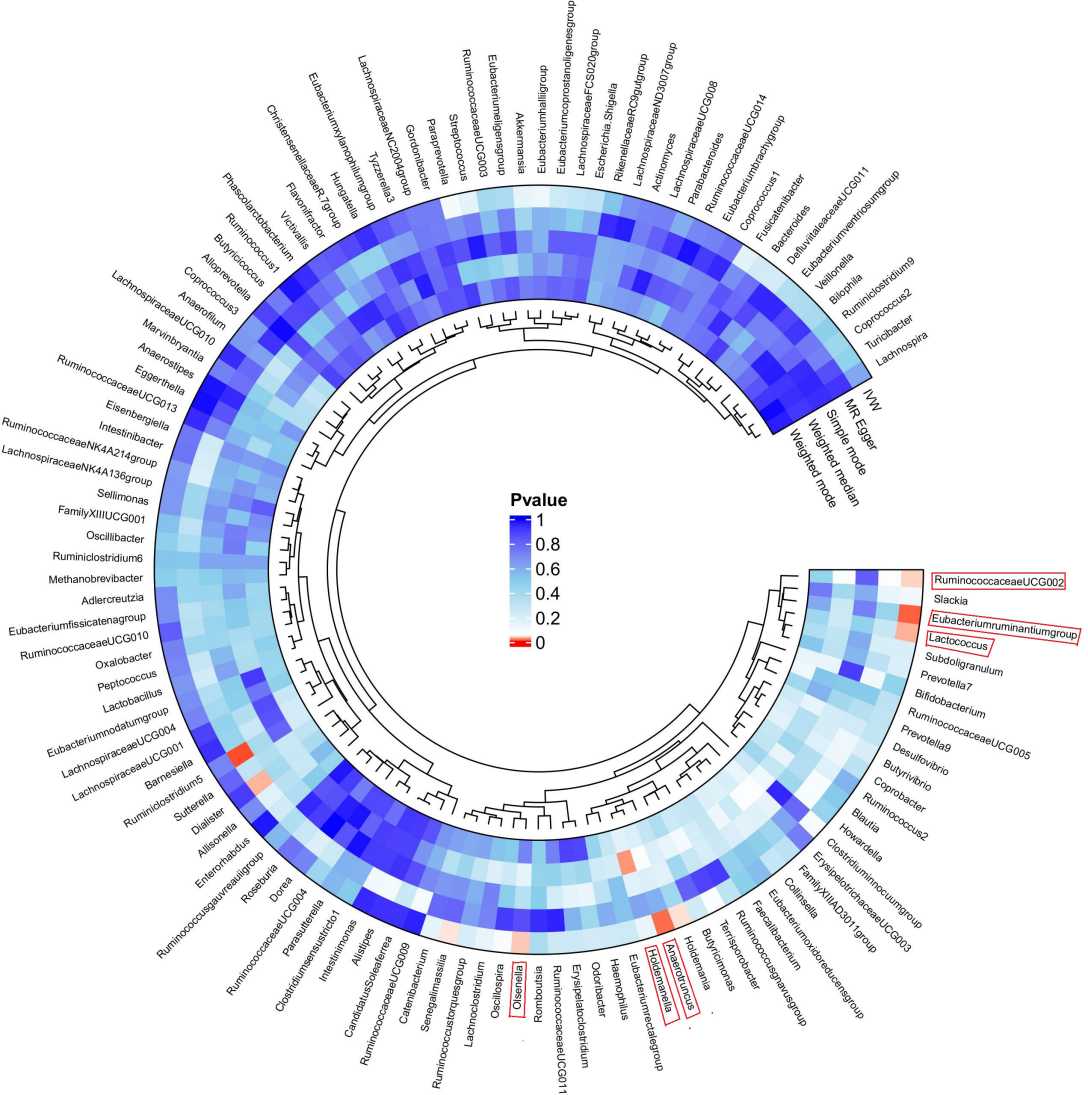
Circular heatmap of MR results across 119 bacterial genera. Each concentric ring represents a different MR method (IVW, MR-Egger, simple mode, weighted median and weighted mode), with colour intensity reflecting *P*-values. The seven genera with nominal IVW associations (*P*<0.05) are highlighted with red/warm-coloured segments in the IVW ring: *Lactococcus*, *Olsenella*, *Senegalimassilia*, Ruminococcaceae UCG-002, *Holdemania*, *E. ruminantium* group and *Anaerotruncus*. None of these genera remained statistically significant after FDR correction.

**Fig. 2.**
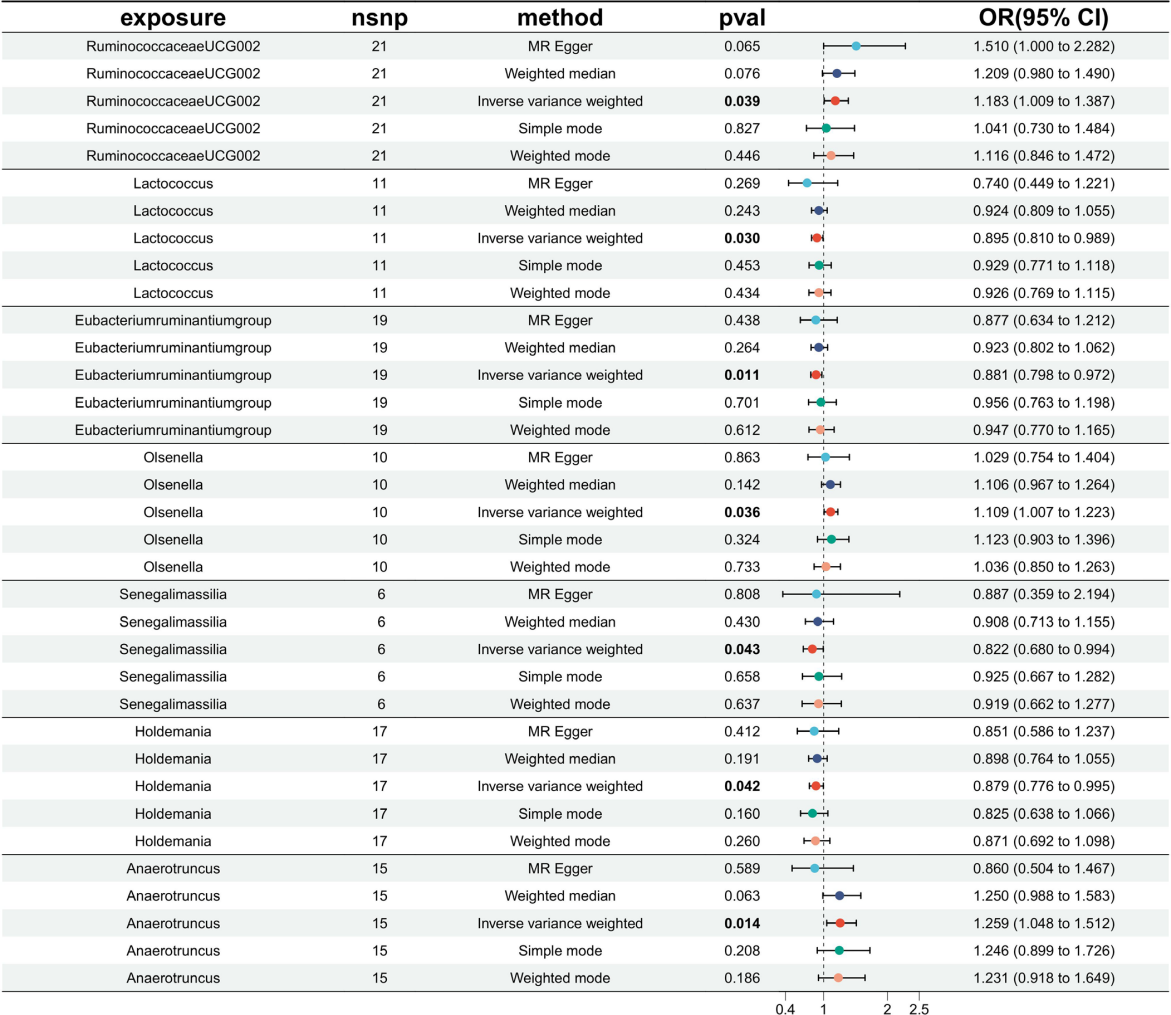
Forest plot of MR results for the seven bacterial genera with nominal IVW associations with endometriosis. Each genus is displayed as a separate group with results for all five MR methods; IVW estimates served as the primary analytical method. The seven genera shown are Ruminococcaceae UCG-002, *Lactococcus*, *E. ruminantium* group, *Olsenella*, *Senegalimassilia*, *Holdemania* and *Anaerotruncus*.

MR analyses of 119 bacterial genera identified seven taxa with nominally significant IVW associations with endometriosis (all nominal *P*<0.05; Fig. 1): *Lactococcus* (OR=0.895, 95% CI: 0.810–0.989, *P*=0.030), *Olsenella* (OR=1.109, 95% CI: 1.007–1.223, *P*=0.036), *Senegalimassilia* (OR=0.822, 95% CI: 0.680–0.994, *P*=0.043), Ruminococcaceae UCG-002 (OR=1.183, 95% CI: 1.009–1.387, *P*=0.039), *Holdemania* (OR=0.879, 95% CI: 0.776–0.995, *P*=0.042), *Eubacterium ruminantium* group (OR=0.881, 95% CI: 0.798–0.972, *P*=0.011) and *Anaerotruncus* (OR=1.259, 95% CI: 1.048–1.512, *P*=0.014). Among these, *Olsenella*, Ruminococcaceae UCG-002 and *Anaerotruncus* were directionally associated with increased endometriosis risk, whereas *Lactococcus*, *Senegalimassilia*, *Holdemania* and *E. ruminantium* group were directionally associated with decreased risk. Detailed results including all five MR methods are presented in Table 1 and Fig. 2.

**Table 1.** MR results of the seven bacterial genera with nominal IVW associations with endometriosis

Gut microbiota	Method	SNP	β	se	*P*-value	Fdr *P*	OR	OR 95% CI lower	OR 95% CI upper
*Lactococcus*	MR Egger	11	−0.301	0.255	0.269	–	0.740	0.449	1.221
	Weighted median	11	−0.079	0.068	0.243	–	0.924	0.809	1.055
	**IVW**	**11**	**−0.111**	**0.051**	**0.030**	**0.731**	**0.895**	**0.810**	**0.989**
	Simple mode	11	−0.074	0.095	0.453	–	0.929	0.771	1.118
	Weighted mode	11	−0.077	0.095	0.434	–	0.926	0.769	1.115
*Olsenella*	MR Egger	10	0.028	0.159	0.863	–	1.029	0.754	1.404
	Weighted median	10	0.100	0.068	0.142	–	1.106	0.967	1.264
	**IVW**	**10**	**0.104**	**0.050**	**0.036**	**0.731**	**1.109**	**1.007**	**1.223**
	Simple mode	10	0.116	0.111	0.324	–	1.123	0.903	1.396
	Weighted mode	10	0.036	0.101	0.733	–	1.036	0.850	1.263
*Senegalimassilia*	MR Egger	6	−0.120	0.462	0.808	–	0.887	0.359	2.194
	Weighted median	6	−0.097	0.123	0.430	–	0.908	0.713	1.155
	**IVW**	**6**	**−0.196**	**0.097**	**0.043**	**0.731**	**0.822**	**0.680**	**0.994**
	Simple mode	6	−0.078	0.167	0.658	–	0.925	0.667	1.282
	Weighted mode	6	−0.084	0.168	0.637	–	0.919	0.662	1.277
*Ruminococcaceae* UCG-002	MR Egger	21	0.412	0.211	0.065	–	1.510	1.000	2.282
	Weighted median	21	0.189	0.107	0.076	–	1.209	0.980	1.490
	**IVW**	**21**	**0.168**	**0.081**	**0.039**	**0.731**	**1.183**	**1.009**	**1.387**
	Simple mode	21	0.040	0.181	0.827	–	1.041	0.730	1.484
	Weighted mode	21	0.110	0.141	0.446	–	1.116	0.846	1.472
*Holdemania*	MR Egger	17	−0.161	0.190	0.412	–	0.851	0.586	1.237
	Weighted median	17	−0.108	0.082	0.191	–	0.898	0.764	1.055
	**IVW**	**17**	**−0.129**	**0.063**	**0.042**	**0.731**	**0.879**	**0.776**	**0.995**
	Simple mode	17	−0.193	0.131	0.160	–	0.825	0.638	1.066
	Weighted mode	17	−0.138	0.118	0.260	–	0.871	0.692	1.098
*E. ruminantium* group	MR Egger	19	−0.131	0.165	0.438	–	0.877	0.634	1.212
	Weighted median	19	−0.080	0.072	0.264	–	0.923	0.802	1.062
	**IVW**	**19**	**−0.127**	**0.050**	**0.011**	**0.731**	**0.881**	**0.798**	**0.972**
	Simple mode	19	−0.045	0.115	0.701	–	0.956	0.763	1.198
	Weighted mode	19	−0.055	0.106	0.612	–	0.947	0.770	1.165
*Anaerotruncus*	MR Egger	15	−0.151	0.272	0.589	–	0.860	0.504	1.467
	Weighted median	15	0.224	0.120	0.063	–	1.250	0.988	1.583
	**IVW**	**15**	**0.230**	**0.094**	**0.014**	**0.731**	**1.259**	**1.048**	**1.512**
	Simple mode	15	0.220	0.166	0.208	–	1.246	0.899	1.726
	Weighted mode	15	0.208	0.149	0.186	–	1.231	0.918	1.649

Note: FDR *P*-values were calculated using the Benjamini–Hochberg method across all 119 genera tested. FDR *P* is reported only for IVW results (primary method). Bold rows indicate primary IVW estimates. None of the seven primary IVW associations survived FDR correction.

After Benjamini–Hochberg FDR correction across all 119 genera, none of the seven associations reached statistical significance at FDR<0.05 (all FDR-adjusted *P*=0.731; Table 1). These results should therefore be interpreted as suggestive, exploratory signals rather than statistically robust associations after multiple testing correction.

### Sensitivity analysis at stricter IV threshold

At a more stringent threshold of *P*<1×10⁻⁵, five of the seven genera retained sufficient independent instruments (≥3 SNPs with F>10): *E. ruminantium* group (14 SNPs), *Anaerotruncus* (10 SNPs), *Lactococcus* (7 SNPs), Ruminococcaceae UCG-002 (15 SNPs) and *Holdemania* (11 SNPs). *Olsenella* and *Senegalimassilia* had insufficient instruments at this threshold and were excluded from this sensitivity analysis. Among the five testable genera, *E. ruminantium* group (OR=0.891, 95% CI: 0.797–0.997, *P*=0.044) and *Anaerotruncus* (OR=1.237, 95% CI: 1.014–1.509, *P*=0.036) maintained consistent effect directions and nominal significance. *Lactococcus* (OR=0.908, *P*=0.078), Ruminococcaceae UCG-002 (OR=1.162, *P*=0.071) and *Holdemania* (OR=0.892, *P*=0.068) showed consistent effect directions but did not reach nominal significance, likely reflecting reduced statistical power with fewer instruments. These threshold-based findings remain exploratory and do not overcome the lack of FDR-significant associations in the primary analysis.

### Heterogeneity and pleiotropy assessments

**Fig. 3.**
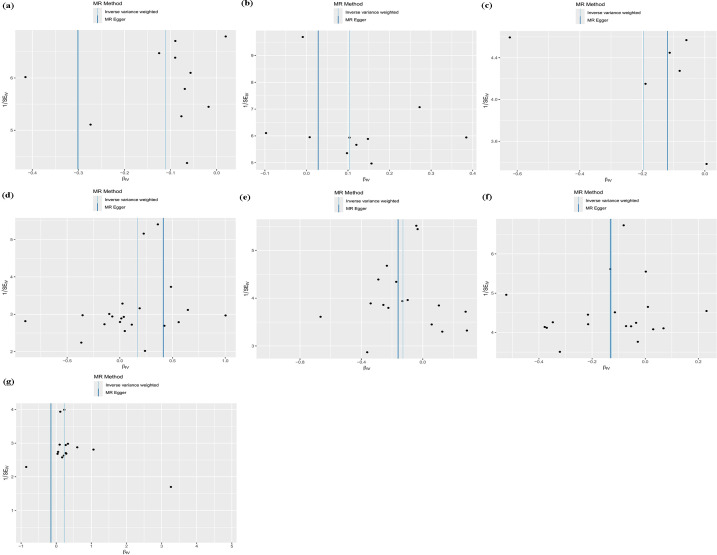
Funnel plots for the seven genera with nominal IVW associations. (a) *Lactococcus*; (b) *Olsenella*; (c) *Senegalimassilia*; (d) Ruminococcaceae UCG-002; (e) *Holdemania*; (f) *E. ruminantium* group and (g) *Anaerotruncus*. The distributions of individual SNP estimates are broadly centred around the IVW point estimate for most genera, although some asymmetry is present in genera with fewer instruments.

**Fig. 4.**
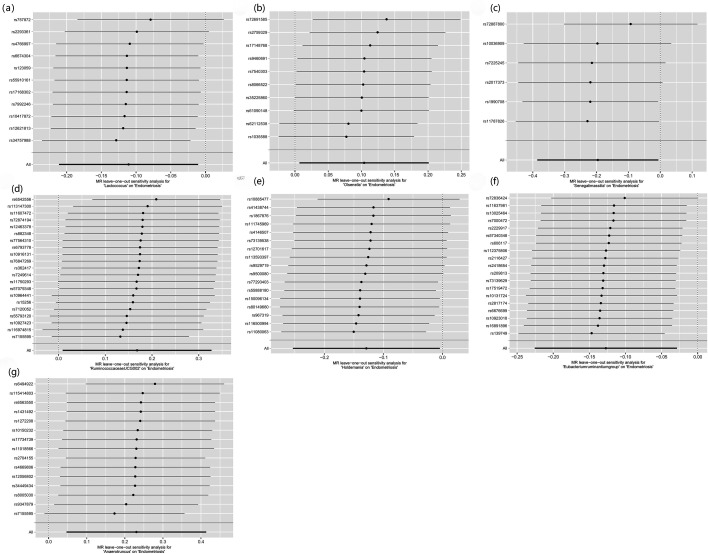
Leave-one-out sensitivity analysis results for the seven genera with nominal IVW associations. (a) *Lactococcus*; (b) *Olsenella*; (c) *Senegalimassilia*; (d) Ruminococcaceae UCG-002; (e) *Holdemania*; (f) *E. ruminantium* group and (g) *Anaerotruncus*.

Cochran’s Q test for heterogeneity and MR-Egger intercept analysis for horizontal pleiotropy detected no significant heterogeneity or directional pleiotropic effects across all seven genera (Table 2). MR-PRESSO global tests were also non-significant for all tested genera. Funnel plots showed that for most genera, the distributions of individual SNP estimates were broadly centred around the IVW point estimate, although some asymmetry was observed for genera with fewer instruments, such as *Senegalimassilia* (6 SNPs; Fig. 3). Leave-one-out sensitivity analyses revealed no substantial influence of any single SNP on the overall effect estimates for any of the seven genera (Fig. 4).

**Table 2.** Tests for heterogeneity and horizontal pleiotropy

Gut microbiota	Heterogeneity Q value	Heterogeneity *P*-value	MR-Egger intercept	Pleiotropy *P*-value
*Lactococcus*	4.789	0.852	0.0254	0.466
*Olsenella*	7.121	0.523	0.010	0.628
*Senegalimassilia*	5.065	0.280	−0.007	0.873
*Ruminococcaceae* UCG-002	26.077	0.128	−0.019	0.225
*Holdemania*	17.832	0.271	0.003	0.861
*E. ruminantium* group	12.732	0.753	0.0004	0.979
*Anaerotruncus*	12.732	0.468	0.028	0.161

## Discussion

This two-sample MR study investigated potential associations between genetically predicted gut microbiota composition and endometriosis risk. Seven bacterial genera – *Lactococcus*, *Olsenella*, *Senegalimassilia*, Ruminococcaceae UCG-002, *Holdemania*, *E. ruminantium* group and *Anaerotruncus* – showed nominally significant associations with endometriosis in IVW analyses. However, none of these associations survived FDR correction for multiple testing across 119 genera, and the findings should be considered suggestive and hypothesis-generating rather than definitive evidence of causality.

Clinical microbiological studies of endometriosis have reported distinct microbial profiles in the reproductive tract and pelvic fluid of affected patients compared with controls. Perrotta *et al*. [23] demonstrated that vaginal microbiota signatures enable endometriosis disease stratification, with *Anaerococcus* exhibiting the highest discriminatory weight. Lee *et al*. [24] revealed altered pelvic fluid microbiota in endometriosis, including increased *Acinetobacter*, *Pseudomonas* and *Streptococcus*. Wessels *et al*. [25] reported increased endometrial microbial diversity in endometriosis patients, a state characterized by shifts in the relative abundances of multiple taxa away from the expected compositional baseline. These observational findings, together with preclinical evidence supporting interplay between gut microbiota and endometriosis pathogenesis [9,26], provide biological context for the nominal associations identified in our MR analysis, but they do not confirm causality.

Among the seven genera identified, some have established biological relevance. *Lactococcus* is a lactic acid-producing genus with documented immunomodulatory properties; its inverse nominal association with endometriosis risk is compatible with evidence suggesting protective roles of lactate-producing bacteria in maintaining mucosal immune homeostasis. *Olsenella*, a member of the Actinobacteria phylum, has been implicated in bile acid metabolism and inflammatory signalling, providing a plausible biological pathway for its positive nominal association with endometriosis. However, several identified genera – including Ruminococcaceae UCG-002, *E. ruminantium* group and *Anaerotruncus* – currently lack strong direct functional or clinical evidence linking them to endometriosis. The observed associations for all seven genera should therefore be interpreted with particular caution and regarded as hypothesis-generating findings warranting further investigation through mechanistic and clinical studies.

A key advantage of the MR approach is its capacity to reduce, although not eliminate, confounding and reverse causation inherent in observational designs. By using genetic variants as proxies for microbial exposures, this framework can provide supportive aetiological evidence when instrument validity, ancestry comparability and statistical power are adequate [10,11]. In the present study, the use of well-characterized GWAS datasets from the MiBioGen consortium and FinnGen, combined with comprehensive sensitivity analyses, improves analytical transparency; nevertheless, the lack of FDR-significant associations substantially limits the strength of inference.

This study has several important limitations that warrant careful consideration. First, the gut microbiota GWAS data from the MiBioGen consortium are derived from a multi-ethnic meta-analysis, whereas the endometriosis GWAS from FinnGen comprises exclusively European-ancestry individuals. This ancestry mismatch is a major limitation and may violate MR assumptions if the genetic architecture of gut microbiota composition differs across populations, potentially introducing bias through population stratification [27]. This limitation cannot be fully addressed through discussion or sensitivity analyses using the currently available summary data; future studies should utilize ancestry-matched GWAS data or perform formal stratified analyses to address this issue. Second, the instrumental variable selection threshold of *P*<5×10⁻⁵ is less stringent than the conventional genome-wide significance level (*P*<5×10⁻⁸). Although this relaxed threshold is widely adopted in gut microbiota MR studies because microbiome GWAS currently identify very few genome-wide significant loci [15], it increases the risk of including pleiotropic or weakly associated variants. Our supplementary analysis at a stricter threshold (*P*<1×10⁻⁵) showed that the two genera with the strongest nominal signals – *E. ruminantium* group and *Anaerotruncus* – retained consistent effect directions and nominal significance, but this sensitivity analysis does not overcome the lack of FDR-significant results in the primary analysis. Third, and critically, the multiple testing correction across 119 genera yielded FDR-adjusted *P*-values of 0.731 for all seven nominally significant genera, indicating that none of the observed associations can be considered statistically significant after accounting for the number of tests performed. Accordingly, the nominal signals may include chance findings and should be treated as exploratory. Fourth, gut microbiota GWAS remains at an emerging stage with constrained sample sizes; the genetic loci identified to date for microbial traits are sparse, which limits statistical power and may result in both false-positive and false-negative findings. Fifth, the MiBioGen consortium characterized microbial composition using 16S rRNA gene sequencing, which provides genus-level but not species-level resolution, potentially masking functionally distinct species within the same genus. Sixth, although MR-Egger intercept analyses did not detect significant horizontal pleiotropy, the possibility of balanced pleiotropy cannot be entirely excluded. Finally, while MR analysis can identify genetic association signals between gut microbiota and endometriosis, establishing definitive biological mechanisms requires further experimental validation through *in vitro* and *in vivo* studies.

## Conclusion

In conclusion, this study identified nominal and suggestive MR-based associations between specific genetically predicted gut microbial genera – *Lactococcus*, *Olsenella*, *Senegalimassilia*, Ruminococcaceae UCG-002, *Holdemania*, *E. ruminantium* group and *Anaerotruncus –* and endometriosis risk. However, none of the observed associations survived FDR correction for multiple testing, and the results should be interpreted as exploratory and hypothesis-generating rather than definitive causal evidence. Replication in larger, ancestry-matched cohorts and functional validation studies are warranted to confirm and extend these preliminary observations.

## Supplementary material

10.1099/jmm.0.002174 Table S1.

## Abbreviations

FDR: false discovery rate
GWAS: genome-wide association study
IVW: inverse-variance weighting
MR: Mendelian randomization
MR-PRESSO: Mendelian randomization pleiotropy residual sum and outlier
SNP: single nucleotide polymorphism
